# Channelling Evolution: Canalization and the nervous system

**DOI:** 10.1371/journal.pbio.0020019

**Published:** 2004-01-20

**Authors:** Jeremy E Niven

## Abstract

A recent paper suggests that genes can interact in networks to limit variation of phenotype. Similar principles might apply to the regulation of ion channels in nerve cells

Individuals within a wild population show remarkably little morphological variation, given the amount of environmental variation they encounter during development and the amount of genetic variation within the population. This phenotypic constancy led to the proposal that individuals were somehow buffered, or canalized, against genetic and environmental variation (Waddington 1942). Clearly, such a mechanism would have important evolutionary consequences; because natural selection acts upon phenotypic variation within a population, canalization first appears to reduce the evolvability of the trait upon which it is acting (Gibson and Wagner 2000). However, canalization also reduces the effects of new mutations (which may be deleterious), potentially allowing individuals to store this genetic variation without suffering the consequences. If canalization breaks down due to genetic or environmental circumstances, then the stored genetic variation will be released, providing an additional substrate for natural selection. In this way, individuals could potentially undergo large, rapid phenotypic changes.

Experiments in both Drosophila and Arabidopsis have suggested that Hsp90 (heat shock protein 90), a member of a family of proteins expressed at high temperatures (heat shock), may be an excellent candidate for bringing about canalization (Rutherford and Lindquist 1998; Queitsch et al. 2002). Several features of Hsp90 suggest that it is an evolutionary buffer, capable of hiding and then releasing genetic variation: (1) individuals heterozygous for mutations in *Hsp83* (the gene encoding Hsp90) show increased levels of morphological abnormalities; (2) individuals treated with a pharmacological inhibitor of Hsp90 show severe morphological abnormalities; (3) the normal function of Hsp90 is to stabilise the tertiary structure of signal transduction molecules involved in developmental pathways; and (4) this function may be compromised by environmental factors, e.g., heat shock.

## Gene Networks Generate Canalization

**Figure 1:**
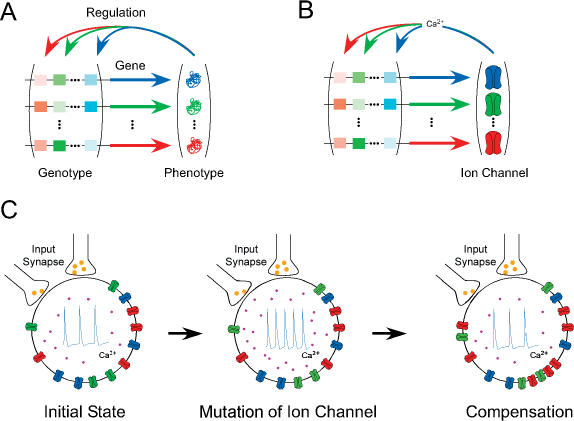
Similarity between a Gene Network Acting as an Evolutionary Buffer and a Gene Network Regulating Neuronal Ion Channel Expression (A) Each gene (horizontal arrow) is regulated by the products of the other genes by means of upstream enhancer elements (boxes). The strength and direction of regulation (depicted as different colour saturation levels) are a function of both the upstream element and the abundance of its corresponding gene product. (B) A similar representation of a putative network for activity-dependent ion channel regulation in a neuron in which Ca^2+^ concentration acts as a feedback mechanism. (C) The mechanism of ion channel compensation in a neuron. The activity of the neuron is dependent upon its synaptic inputs and the suite of ion channels it expresses. Mutation of a gene encoding an ion channel leads to a change in the properties of that channel (depicted as a change in colour saturation) and hence to an increase in activity and internal Ca^2+^ (purple). These changes induce a compensatory increase in the expression of another ion channel (red) to restore the original level of activity.

Hsp90 may not, however, be uniquely placed to act as an evolutionary buffer producing canalization. Recent theoretical work has suggested that canalization may be an emergent property of complex gene networks and may not require specific mechanisms of protein stabilisation and environmental coupling such as those provided by Hsp90 (Siegal and Bergman 2002). Siegal and Bergman (2002) proposed that when a network is compromised by ‘knocking out’ one of several genes, buffering may be lost or compromised, releasing variation that was hidden in the intact network. To test this, Bergman and Siegal (2003) used numerical simulations of a complex network of ten genes in which each gene is capable of influencing the expression of other genes as well as itself (Figure 1A). This network essentially defines the genotype of the individuals within the population, and the amount of gene expression at equilibrium defines the phenotype. Comparison of populations founded by either wild-type individuals or those with a single gene ‘knockout’ revealed much higher levels of phenotypic variation in populations derived from the ‘knockouts’.

Thus, populations derived from ‘knockouts’ express phenotypic variation that was not expressed by the wild-type network, suggesting that any of the genes within the network may buffer genetic variation. This suggests that at least one aspect of generating evolutionary buffering is not unique to Hsp90. But can genes that, unlike Hsp90, are not conditional upon the environment act as evolutionary buffers? To test this, Bergman and Siegal (2003) simulated a gene network that incorporated a mutation process in which single genes may be ‘knocked out’ and then, at a later time, restored. The simulated populations were allowed to evolve whilst being selected for an optimum phenotype (i.e., the populations were exposed to an environment in which a particular phenotype was optimal). A new optimum phenotype was then specified in which the expression of three of the ten network genes changed from on to off or vice versa (i.e., there was a shift in the environmental conditions favouring a different phenotype). Populations evolving with the mutation process reached the new optimum before populations without the mutation process. Thus, the ‘knockout’ mutations were clearly beneficial because they sped up adaptation to a new phenotypic optimum by releasing hidden genetic variation, thereby providing a new substrate upon which natural selection may act. Yet these mutations were not coupled to the new environment, suggesting that the release of the hidden genetic variation does not have to be linked to an environmental change in order to be beneficial.

The simulations described by Bergman and Siegal (2003) suggest that the key properties of an evolutionary buffer, the ability to store and then release genetic variation in response to environmental or genetic change, are not unique to Hsp90. Indeed, the simulations suggest that evolutionary buffering may be a widespread property of gene networks. They also suggest that the hidden genetic variation does not have to be revealed by an environmental change, but can be produced by a gene ‘knockout’. These results may go some way to explain the original observation by Waddington (1942) of phenotypic constancy, yet many questions remain (Stearns 2003). One of the major outstanding questions must be whether it is possible to verify these results experimentally. Bergman and Siegal (2003) used data from the yeast Sacchromyces cerevisiae, in which each gene may be ‘knocked out’ in turn and the expression of the remaining genes determined, to demonstrate that their simulations also had application to biological gene networks. Using these data, they showed that ‘knockouts’ show greater variability in gene expression than wild-type yeast, suggesting that buffering has been disrupted.

## Ion Channels as Evolutionary Buffers

Given the results of Bergman and Siegal (2003), it should be possible to find gene networks in which the elimination of single genes reveals variation in gene expression and hence in phenotype. One class of gene network that may conform to the structure outlined by Bergman and Siegal (2003) is that of the gene networks regulating ion channel expression in neurons. Neurons contain an array of voltage-dependent Na^+^ and K^+^ channels as well as numerous Cl^−^, Ca^2+^, and voltage-independent leak channels. The electrical properties of a single neuron are dependent, though not exclusively, upon the suite of ion channels expressed within that neuron. The properties of a neural network, which generates behaviour, are determined both by the intrinsic expression patterns of ion channels within neurons and the connectivity between neurons. The nervous system develops as an interaction between experience and genetically programmed events. One mechanism by which this interaction is achieved is ion channel compensation (Turrigiano 1999); individual neurons can change their sensitivity to inputs by altering the relative proportion of ion channels, enabling them to maintain stable properties in the face of changing experience (Turrigiano et al. 1994; Brickley et al. 2001; Maclean et al. 2003; Niven et al. 2003a) (Figure 1B).

Many studies of ion channel ‘knockouts’ show relatively little change in overall neuronal activity, although predictions based upon pharmacological blockade of the ion channels suggest there should be a more severe phenotypic change (Marder and Prinz 2002). Subsequent work has shown that the loss of an ion channel may often be compensated by a change in the expression of other ion channels. For example, the neurons upon which I work are Drosophila photoreceptors. In these neurons, loss of the one particular ion channel leads to compensatory changes in other ion channels linked to the activity of the neuron to restore the ability to process visual information (Niven et al. 2003a, 2003b). However, these changes do not restore the original phenotype completely, and the compensated photoreceptors still show a reduced ability to process visual information. In many neurons, it appears that the intracellular Ca^2+^ concentration acts as an internal sensor of neural activity (Marder and Prinz 2002). Ca^2+^, along with other second messengers, may influence the expression of genes encoding ion channels, allowing their expression to be coupled to neural activity (Berridge 1998) (Figure 1B and 1C). Additionally, activity-independent mechanisms of ion channel compensation have been described in which the expression of one ion channel is linked to the expression of other opposing ion channels within a neuron (Maclean et al. 2003). These two systems of activity-dependent and activity-independent ion channel compensation bear a close resemblance to the gene network simulated by Bergman and Siegal (2003) in which each gene regulates its own expression and that of other network genes.

It is possible, therefore, that the networks of genes regulating ion channel expression may act as evolutionary buffers. The relationship between neural activity and the network of ion channel encoding genes may stabilise the neural activity in relation to both the genetic and environmental variation. The stabilisation of neural activity may have consequences for the generation of adaptive behaviour, which is constructed from neural activity. It is possible that ion channels could canalize the evolution of the nervous system by reducing behavioural variation and therefore removing the substrate on which natural selection may act. For example, changes in voltage-dependent Na^+^ channel properties (such as the activation voltage) may be compensated for by regulating the expression of other ion channels. ‘Knockout’ of one of these compensating ion channels may reveal the change in voltage-dependent Na^+^ channel properties, resulting in a shift in the output of the neuron. This hypothesis has several testable predictions. For example, ‘knocking out’ an ion channel should increase the variation in the activity of particular neurons among individuals in a population. This variation in neural activity may produce an effect on the behaviour of the whole organism.

Studying canalization in ion channel gene networks may have significant advantages over studying developmental gene networks because it is relatively straightforward to measure the amounts of ion channels expressed in single identified neurons, to alter the expression of individual ion channels, and to relate these alterations to behaviour. I am currently pursuing the impact of ion channel compensation in Drosophila photoreceptors (Niven et al. 2003a, 2003b, 2003c). In this system, changes in ion channel expression produce changes in the coding of visual information, which may lead to behavioural differences. The possible role of ion channel compensation in canalizing the evolution of the nervous system may have important implications not just for understanding this system, but also for understanding the contribution of ion channel compensation to the function of the nervous system and its evolution.

## Abbreviation

Hsp90: heat shock protein 90
